# Supplementary material for: The Effects of Long-Term High Fat and/or High Sugar Feeding on Sources of Postprandial Hepatic Glycogen and Triglyceride Synthesis in Mice
Source: Nutrients. 2024 Jul 9;16(14):2186. doi: 10.3390/nu16142186 (PMC11279633; doi:10.3390/nu16142186)
Supplement: Supplementary file 1 [file nutrients-16-02186-s001.zip › nutrients-3087982-supplementary.pdf]

Supplementary data

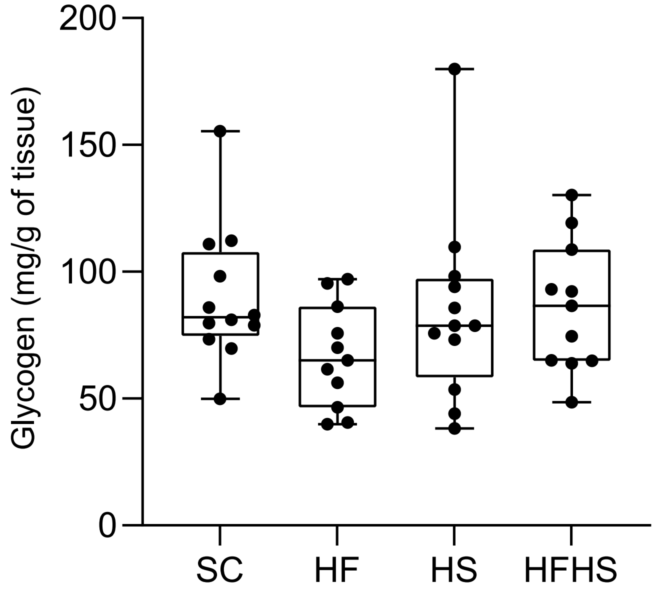

Figure S1: Hepatic glycogen concentrations measured after overnight feeding in mice fed standard chow (SC, *n*=12), high-fat chow (HF *n*=11), standard chow supplemented with glucose/fructose in the drinking water (HS *n*=12), and high-fat chow diet supplemented with glucose/fructose in the drinking water (HFHSD *n*=11).

Table S1: Proximate composition and principal lipid and carbohydrate components of standard chow (SC, Mucedola s.r.l. 223426) and a personalized high-fat (HF, Mucedola s.r.l. 223425) chow diets.

**Parameters**	**Components**	**SC**	**HF**
**Proximate composition**	Protein (% by weight)	19	25
	Lipids (% by weight)	4	30
	Carbohydrate (% by weight)	71	38
	Fiber (% by weight)	2	3
	Ash (% by weight)	4	4
**Specific components**	Starch (% of total carbohydrate)	92	92
	Sucrose (% of total carbohydrate)	8	8
	Polyunsaturated (% of total lipids)	59	59
	Monounsaturated (% of total lipids)	24	24
	Saturated (% of total lipids)	17	17

Table S2: Partial fatty acid compositional analysis of liver triglyceride. This includes the relative abundances of fatty acid moieties quantified from their ^{13}C-NMR ω-2 signals: total saturated fatty acids, oleic (OL), palmitoleic (PO) and linoleic (LO) fatty acids, and total ω-3 fatty acids quantified from the ^{1}H-NMR terminal methyl signals. These were obtained from mice fed standard chow (SC), high-fat chow (HF), normal chow supplemented with HFCS-55 formulation in the drinking water (HS) and high-fat chow supplemented with HFCS-55 formulation in the drinking water (HFHS). Data are shown as means accompanied by standard deviations in parentheses. For each parameter column, significant differences in values between the four diets are denoted by different superscript letters.

**Diet**	**Fatty acid species**				
	**Total Saturated (%)**	**OL (%)**	**PO (%)**	**LO (%)**	**Total ω-3 (%)**
**SC**	36 (6) ^{a}	40 (5) ^{a}	14 (2) ^{a}	6 (2) ^{a,c}	4 (2) ^{a,c}
**HF**	30 (2) ^{b}	35 (5) ^{a}	6 (1) ^{b}	24 (4) ^{b}	6 (2) ^{a}
**HS**	30 (2) ^{b}	52 (2) ^{b}	14 (2) ^{a}	2 (1) ^{a}	2 (1) ^{b}
**HFHS**	32 (4) ^{a,b}	43 (4) ^{a}	7 (1) ^{b}	13 (4) ^{b,c}	3 (1) ^{b,c}

Table S3: The fractional distribution of saturated fatty acids (**SFA**), monounsaturated fatty acids (**MUFA**) and linoleic acid (**LO**) in the glycerol *sn*1,3 and *sn*2 positions of hepatic triglyceride resolved by their ^{13}C-NMR signals for mice fed standard chow (**SC, *n*=10),** high-fat chow (**HF *n*=7**), standard chow supplemented with fructose/glucose in the drinking water (**HS, *n*=12**), and high-fat chow diet supplemented with glucose/fructose in the drinking water (**HFHS, *n*=10**). The triglyceride *sn*1,3 region also contained a resonance of minor intensity that could not be assigned to the other fatty acid classes (Unknown) but was included in the total sum of *sn*1,3 signals for calculating the fractional abundance of the known fatty acid species. Data are shown as means accompanied by standard deviations in parentheses. For each parameter column, significant differences in values between the four diets are denoted by asterisks or superscript letters, accordingly.

Diet	Glycerol *sn*2 position			Glycerol *sn*1,3 positions			
	**SFA**	**MUFA**	**LO**	**SFA**	**MUFA**	**LO**	**Unknown**
**SC**	0.08 (0.05)	0.73 (0.05)	0.20 (0.06)	0.46 (0.04)****	0.39 (0.03)****	0.04 (0.02)****	0.11 (0.03)
**HF**	0.03 (0.01)	0.44 (0.09)	0.54 (0.08)	0.43 (0.01)***	0.35 (0.04)[a]	0.16 (0.05)***	0.06 (0.02)
**HS**	0.04 (0.01)	0.88 (0.04)	0.08 (0.03)	0.41 (0.02)****	0.39 (0.04)****	0.04 (0.02)***	0.15 (0.03)
**HFHS**	0.04 (0.02)	0.62 (0.09)	0.34 (0.10)	0.45 (0.04)****	0.37 (0.03)****	0.09 (0.05)****	0.08 (0.04)

**** $p < 0.0001$ compared to fraction in *sn*2 position

*** $p < 0.001$ compared to fraction in *sn*2 position

[a] $p = 0.053$ compared to fraction in *sn*2 position

Table S4: ^2H and ^{13}C excess enrichments of liver triglyceride from mice fed standard chow (**SC, *n*=12**), high-fat chow (**HF, *n*=11**), normal chow supplemented with 55/45 fructose/glucose at 30 % (w/v) in the drinking water (**HS, *n*=12**) and high-fat chow supplemented with 55/45 fructose/glucose at 30 % w/v in the drinking water (**HFHS, *n*=11**). Data are shown as means accompanied by standard deviations in parentheses.

**Diet**	**^2H-Enrichment (%)**				**^{13}C-Enrichment (%)**	
	Body water	***Glycerol H1, H3***	***Fatty acid CH_3***	***Fatty acid α-CH_2***	***Glycerol C1,C3***	***Fatty acid CH_3***
**SC**	4.86 (0.43)	2.43 (0.38)	0.85 (0.32)	1.23 (0.30)	N.D.	N.D.
**HF**	5.93 (0.76)	1.93 (0.63)	0.27 (0.04)	0.43 (0.08)	N.D.	N.D.
**HS**	5.25 (0.39)	1.58 (0.33)	0.75 (0.22)	1.00 (0.27)	2.49 (0.86)	0.56 (0.14)
**HFHS**	6.14 (0.20)	1.37 (0.35)	0.47 (0.12)	0.54 (0.12)	1.24 (0.68)	0.24 (0.15)

Table S5: ^{2}H excess enrichments of liver glycogen in hydrogens 1-6$_s$ (H1-H6$_S$) from mice fed normal chow (CTL), high-fat chow (HF), normal chow supplemented with 55/45 fructose/glucose at 30 % (w/v) in the drinking water (HS) and high-fat chow supplemented with 55/45 fructose/glucose at 30 % w/v in the drinking water (HFHS). Also shown is the theoretical hydrogen 2 enrichment after correction for incomplete glucose-6-phosphate-fructose-6-phosphate exchange (H2$_{corr}$). Data are shown as means accompanied by standard deviations in parentheses.

**Diet**		**Positional glycogen ^{2}H-enrichments (%)**						
	Body water	***H1***	***H2***	***H2$_{corr}$***	***H3***	***H4***	***H5***	***H6$_S$***
**CTL (n=12)**	4.86 (0.43)	1.44 (0.19)	3.47 (0.41)	5.30 (0.47)	1.06 (0.14)	1.48 (0.17)	1.57 (0.21)	1.02 (0.16)
**HF (n=11)**	5.93 (0.76)	2.80 (0.30)	4.47 (0.57)	6.84 (0.87)	2.72 (0.40)	3.13 (0.35)	3.38 (0.34)	2.56 (0.29)
**HS (n=12)**	5.25 (0.39)	1.12 (0.40)	3.73 (0.77)	5.71 (1.18)	1.63 (0.41)	1.92 (0.44)	1.98 (0.44)	0.86 (0.41)
**HFHS (n=11)**	6.14 (0.20)	1.70 (0.30)	4.20 (0.29)	6.43 (0.44)	2.05 (0.22)	2.50 (0.27)	2.49 (0.22)	1.30 (0.32)

Table S6: ^{13}C excess enrichments of glycogen carbons 2 and 5 and selected ^{13}C-isotopomers from mice fed normal chow supplemented with 55/45 fructose/glucose at 30 % w/v in the drinking water (HS) and high-fat chow supplemented with 55/45 fructose/glucose at 30 % w/v in the drinking water (HFHS). The fructose component was enriched to 20 % with [U-^{13}C]fructose on the ultimate night of the 18-week feeding trial. Data are shown as means accompanied by standard deviations in parentheses.

**Diet**	**Glycogen ^{13}C-excess enrichment (%)**		**Glycogen ^{13}C-isotopomer enrichment**		
	Carbon 2	***Carbon 5***	***[4,5,6-$^{13}C_3$]***	***[1,2-$^{13}C_2$]***	***[5,6-$^{13}C_2$]***
**HS (n=12)**	3.37 (0.97)	3.15 (1.22)	2.98 (1.15)	0.49 (0.14)	0.11 (0.05)
**HFHS (n=11)**	2.59 (0.65)	2.11 (0.80)	1.94 (0.72)	0.42 (0.08)	0.11 (0.06)
